# Supplementary material for: Qualitative systems mapping for complex public health problems: A practical guide
Source: PLoS One. 2022 Feb 25;17(2):e0264463. doi: 10.1371/journal.pone.0264463 (PMC8880853; doi:10.1371/journal.pone.0264463)
Supplement: S1 File — Interview guide for the international expert interviews. This interview data is here used to illustrate our methodology and has been published elsewhere [19]. (PDF) [file pone.0264463.s001.pdf]

Interview guide

Instructions to the interviewer

Instructions to the interviewer are in italics.
Text to be read is in bold.

If a respondent declines to answer a question, please write 'declined to answer' in the margin.

Information of the study

Hello, I am …. from …. Working on a research project concerning HIV drug resistance. By interviewing experts from several fields, I aim to construct a broad overview of all possible factors leading to HIV drug resistance and possible solutions to these problems.
You have been selected for this interview because of your expertise in X. Before we start I would like to remind you that the information you provide is completely confidential. Your responses are recorded but this questionnaire does not have your name on it and is only identified by a number. If you have questions after we are finished, you can always contact me.

Interview information

Interview number:

Date of interview:

Interviewer name:

Location:

Time of start:

Language:

Part 1: Sociodemographic information

1.1 Gender of respondent

☐ Male
☐ Female

☐ Other

If you don't mind, I would like to start by asking you some questions about your age, educational background and work.

1.2 How old are you?

1.3 What did you study?

1.4 When did you graduate?

Part 2: HIV related experience

2.1 Which work are you doing at the moment and how is it related to HIV or HIV drug resistance?

2.2 In which SSA countries have you worked before and are you working at the moment?

2.3 Are you active in an association concerning Sub Saharan Africa or HIV beyond your job or intersecting with your job?

Part 3:

3.3 In your experience, what are the main causes of HIV drug resistance?
The following four areas should be covered:

☐ Availability of ART at local healthcare center

☐ PLHIV fetches / is able to fetch his/her ART

☐ PLHIV takes the ART as prescribed

☐ ART suppresses the viral load

Reasons outside these four areas:
3.4 What do you think is causing the problems/situations you just mentioned?

3.5 Can you think of some solutions for the problems you have just mentioned? These can be your personal ideas or solutions that have already been studied and implemented.

This concludes our interview. Thank you very much for your participation.

Time of conclusion:

Additional interviewer notes:
