# Supplementary material for: Qualitative systems mapping for complex public health problems: A practical guide
Source: PLoS One. 2022 Feb 25;17(2):e0264463. doi: 10.1371/journal.pone.0264463 (PMC8880853; doi:10.1371/journal.pone.0264463)
Supplement: S2 File — Interview guide for the interviews with PLHIV in Dar es Salaam, Tanzania. This interview data is here used to illustrate our methodology and has been published elsewhere [20]. (PDF) [file pone.0264463.s002.pdf]

# Interview guide for PLHIV

## English Version

Interview guide (People living with HIV)
English Version
Instructions to the interviewer

*Instructions to the interviewer are in italics.*
*Text to be read is in bold.*

*If a respondent declines to answer a question, please write 'declined to answer' in the margin.*

Information of the study

**Hello, my name is ….. I am coming from ….. working in a collaborative project with ….. working on a research project concerning HIV drug resistance. By interviewing people living with HIV, this research will construct a broad overview of all possible factors leading to HIV drug resistance and possible solutions to these problems.**

**You have been selected for this interview because you are living with HIV. Before we start I would like to repeat some items from the informed consent form:**

**The information you provide is completely confidential. Your responses are recorded but this questionnaire does not have your name on it and is only identified by a number.**
**If you have questions after we are finished you can always contact me.**

Interview information
Interview number:

Date of interview:

Interviewer name:

Location:

Time of start:

Language:

Part 1: Sociodemographic information

**I. Baseline interviewee profile:**

| | |
|---|---|
| A1 | Sex (Gender) of interviewee: [ ] Male [ ] Female |

| | |
|---|---|
| A2 | Age (in years)............. |
| A3 | Religion: |
| A4 | Marital status: |
| A5 | Job: |
| A6 | Level of education or degrees: |
| | Year when highest level of education was completed |
| A7 | May we contact you if we have any additional questions in the weeks/months ahead? [ ] Yes [ ] No If not, why? |
| A8 | **If yes** Contact Information:<br>Phone: Email: |

Part 2: HIV related background

**2.1 When did you first learn about your HIV positive status?**
**and when did you start taking antiretroviral therapy?**

**2.2 Which medication are you taking?**
**and which line of treatment are you on?**
**2.2.1 Do you use other types of HIV treatment, next to the medication you are taking? If yes can you explain about them?**

**2.3 How often do you have to visit the healthcare center in relation to your HIV status?**
**2.3.1 Do you feel like you get the necessary counselling from your healthcare provider? Please explain**

**2.4 Are you a member of any peer support group or other organization concerning HIV support or advocacy? If yes please explain**

Part 3:
**3.3 Could you describe some of the challenges you face concerning your HIV treatment?**
*The following four areas should be covered:*

Availability of ART at your local healthcare center (this aims to cover drug stock-outs)

 **Your** ability to fetch your ART supplies (this aims to cover all the reasons people would not be able to / want to go to the healthcare center for ART pick up or follow-up visits)

 **Your adherence in** taking the ART as prescribed (this aims to cover all the reasons why people don’t always take their medication daily and as prescribed)

3.3.1 Do you feel like the medication you are taking is helping you?  If yes explain, if no explain

*Reasons outside these four areas:*
**3.4 What do you think are the causes of the problems/situations you just mentioned?**
*Go deeper into some of the issues indicated by the interviewee in the previous questions.*

**3.5 What would help you to take your treatment daily as prescribed?**
*Here other solution-oriented questions can be asked depending on the previous course of the interview. For example: "What would be the best way to overcome stigma?".*

**3.6 Is there anything else you think is relevant for our discussion that we haven't discussed so far?**

**This concludes our interview. Thank you very much for your participation.**

*Time of conclusion:*

*Additional interviewer notes:*
