# Supplementary material for: Qualitative systems mapping for complex public health problems: A practical guide
Source: PLoS One. 2022 Feb 25;17(2):e0264463. doi: 10.1371/journal.pone.0264463 (PMC8880853; doi:10.1371/journal.pone.0264463)
Supplement: S3 File — Interview guide for the local actors in Dar es Salaam, Tanzania. This interview data is here used to illustrate our methodology and has been published elsewhere [20]. (PDF) [file pone.0264463.s003.pdf]

Interview guide for local actors

English Version

Instructions to the interviewer

Instructions to the interviewer are in italics.
Text to be read is in bold.

If a respondent declines to answer a question, please write ‘declined to answer’ in the margin.

Information of the study

Hello, my name is … I am coming from … working in a collaborative project with … working on a research project concerning HIV drug resistance. By interviewing experts from several fields, this research will construct a broad overview of all possible factors leading to HIV drug resistance and possible solutions to these problems.

You have been selected for this interview because of your role in [study site] as a …X. Before we start I would like to repeat some items from the informed consent form:

The information you provide is completely confidential. Your responses are recorded but this questionnaire does not have your name on it and is only identified by a number.

If you have questions after we are finish the interview you can always contact me.

Interview information
Interview number:

Date of interview:

Interviewer name:

Location:

Time of start:

Language:

Part 1: Sociodemographic information

I. Baseline interviewee profile:

A1	Job:
A3	Sex (Gender) of interviewee: [ ] Male [ ] Female
A4	Age (in years).............
A5	Months/years of experience in current job:
A6	Level of education:
A7	Where where you born?
A8	Where do you live at the moment?
A9	May we contact you if we have any additional questions in the weeks/months ahead? [ ] Yes [ ] No If not, why?
A10	If yes, you contact information:<br>Phone: Email:

Part 2: HIV related experience

2.1 Could you describe what you are doing in your work? What are your daily activities and responsibilities?

2.2 Do you encounter PLHIV at your work?
2.2.1 If yes, how often are you in contact with them?
2.2.2 What is the nature of your contact with PLHIV? ***(logistic, medical, psychosocial,…)***
2.2.3 Do you give assistance to PLHIV? If yes, which type of assistance? If no, please explain

Part 3:
3.3 In your experience, what are the issues PLHIV face in their daily life?
The following four areas should be covered:

 Concerning the availability of ART at the local healthcare center

 Concerning picking up their ART at the healthcare center

 Concerning the ability to take the ART every day as prescribed

 Concerning the effect of the medication (*capturing side effects but also believes about the medication)*

Reasons outside these four areas:

3.4 What do you think is the cause of ………………………………………………..?
(Go deeper into some of the problems described above)

3.5 Have you ever heard about HIV drug resistance?
3.5.1 If yes, what do you know about HIV drug resistance?
3.5.2 Where did you hear this information?

3.6 What do you think the people living with HIV in your community need?
(Adapt the question based on the previous discussion. E.g. What do you think the PLHIV in your community need to overcome stigma? What do you think would help them to take their medication correctly,…)

3.6 Do you have anything important to our discussion that you want to share with us?

This concludes our interview. Thank you very much for your participation.
Time of conclusion:
Additional interviewer notes:
